# Deep Learning-Based Automated Anatomical Landmark Detection and Saw Blade Size Prediction for Canine Tibial Plateau Leveling Osteotomy

**DOI:** 10.3390/ani16111599

**Published:** 2026-05-24

**Authors:** Tea Hyung Kim, Ji Yun Lee, Hwi Yool Kim

**Affiliations:** 1 Department of Veterinary Medicine, Konkuk University, Seoul 05029, Republic of Korea; tim4546@naver.com; 2 Digital Strategy Team, Chung-Ang University Healthcare System, Seoul 06973, Republic of Korea; 19784@cauhs.or.kr

**Keywords:** dog, TPLO, tibial plateau angle, radiography, deep learning, landmark detection, preoperative planning, veterinary orthopedics

## Abstract

Objective: To develop and validate a fully automated deep learning workflow that localizes key anatomical landmarks on standard canine hindlimb lateral radiographs, derives the tibial plateau angle (TPA), and recommends a saw blade size for tibial plateau leveling osteotomy (TPLO) preoperative planning. Study Design: Retrospective validation study. Animals: Two hundred annotated lateral radiographs obtained from 130 dogs representing 14 breeds, with body weights ranging from 2.4 to 38.0 kg. Methods: A customized four-stage U-Net was trained using three complementary grayscale representations (normalized, contrast-enhanced, and gamma-adjusted images) to detect five TPLO-related landmarks. A deterministic geometric module then calculated TPA and mapped the derived osteotomy geometry to the nearest clinically available saw blade class. Results: The mean absolute error for TPA prediction was 1.34 ± 1.73°, and the median absolute error was 0.75°. Overall, 164/200 cases (82.0%) were within 2° and 188/200 cases (94.0%) were within 4.8° of the surgeon reference. Mean bias was −0.39°, the 95% limits of agreement ranged from −4.62° to 3.85°, and Pearson’s correlation coefficient was 0.87. For saw blade size prediction, mean absolute error was 0.32 ± 0.85 mm, exact agreement was achieved in 175/200 cases (87.5%), and all predictions remained within one adjacent class. Conclusions: The proposed pipeline provided clinically useful automated estimates of TPA and saw blade size from routine lateral radiographs. However, occasional high-impact landmark failures remained, indicating that the system should be positioned as an interpretable decision-support tool that requires surgeon verification rather than as an unsupervised autonomous planning system.

## Simple Summary

Planning for tibial plateau leveling osteotomy (TPLO) relies on measurements obtained from a single lateral radiograph, but those measurements are still affected by manual landmark placement and therefore remain observer-dependent. In this study, we combined a U-Net-based landmark detector with a deterministic geometric module to automate tibial plateau angle (TPA) measurement and saw blade size selection in 200 canine radiographs from 130 dogs, encompassing both unilateral and bilateral stifle evaluations. The study population comprised 14 breeds across a body weight range of 2.4 to 38.0 kg, with small-breed dogs (body weight ≤ 5 kg) accounting for 49.5% of cases and medium-breed dogs (body weight 5–15 kg) accounting for 49.5% of cases. Across the full cohort, the mean absolute error for TPA was 1.34 degrees; 164 of 200 cases (82.0%) were within 2 degrees of the surgeon reference, and 188 of 200 cases (94.0%) were within 4.8 degrees, a commonly cited range for interobserver measurement variability. Exact saw blade size agreement was achieved in 175 of 200 cases (87.5%), and all predictions remained within one adjacent clinical size class. Although the median error was low for all five landmarks, rare large failures in axis-related landmarks indicate that automated plans should still be reviewed by the surgeon before clinical use.

## 1. Introduction

Cranial cruciate ligament rupture is a commonly found orthopedic disorder in dogs that requires surgical treatment, and tibial plateau leveling osteotomy (TPLO) is widely used to restore functional stability of the stifle joint [1]. Because the site and degree of correction are determined based on radiographic geometry rather than direct intraoperative visualization of the biomechanical target, meticulous preoperative planning is essential [1,2].

The tibial plateau angle (TPA) is a fundamental element in TPLO planning [1,2]. Typically, the TPA is measured on a lateral hindlimb radiograph by establishing the tibial functional axis and the tibial plateau axis, both of which rely on the manual identification of anatomical landmarks [2,3]. Consequently, the measured angle can be affected by operator experience, radiographic positioning, and projection geometry [2,3,4,5,6]. Classic radiographic studies have reported interobserver variability of about 4.8 degrees and intraobserver variability of about 3.4 degrees [2,3,4,5,6].

This variability in manual landmark placement influences the planning of saw blade size. Digital planning tools are more efficient than analog templates; however, they still rely on human judgment to identify anatomical points [6,7,8]. A system that automatically localizes anatomical structures and subsequently applies transparent geometric rules may enhance reproducibility while maintaining interpretability [9,10,11].

Deep learning is progressively more valuable in veterinary diagnostic imaging for tasks such as thoracic radiograph classification [12], detection of cardiomegaly [13,14], screening for left atrial enlargement [15], assessment of pulmonary edema [16], diagnosis of stifle disease [17], grading of tracheal collapse [18], screening for elbow dysplasia [19], and analysis of the hip or Norberg angle [20,21,22]. Comprehensive reviews of artificial intelligence applications in veterinary diagnostic imaging are available elsewhere [23,24,25]. In human diagnostic imaging, heatmap-based landmark localization and geometry-aware modeling have also shown strong performance, especially when learned localization is integrated with explicit spatial reasoning [9,10,26]. Nonetheless, reports of TPLO-specific integrated workflows that automatically identify landmarks and then derive both TPA and saw blade size remain limited.

In TPLO planning, the significance of these measurements extends far beyond mere numerical accuracy, as they are fundamental to surgical success and joint stability. Minor angular deviations may alter the intended amount of plateau rotation, and small size mismatches could shift the osteotomy arc, thereby affecting the tibial tuberosity width, fragment geometry, and the workspace for the implant [1,8,27]. These issues are especially important in toy-breed or small-breed dogs, or in tibias with a narrow tibial tuberosity margin [1,8,27]. Additionally, breed conformation and body size may influence the definition and visibility of anatomical landmarks on radiographs, as morphological differences across breeds can alter the shape and relative position of the proximal tibia.

Accordingly, a clinically useful automated system should not only achieve an acceptable average error but should also generate outputs that can be traced and audited when disagreement occurs [11]. For this reason, we adopted a hybrid design. Rather than directly regressing TPA or saw blade class from the image as a black box, deep learning was used only for landmark localization, whereas all subsequent measurements remained deterministic and geometry-based [9,10]. This design aligns with the wider emphasis on developing interpretable and reviewable clinical artificial intelligence systems that serve to support, rather than replace, expert judgment [11,23,25].

The objective of the current study was to develop and validate a deep learning pipeline for the automatic identification of five TPLO-related landmarks on canine lateral radiographs and to combine those predictions with a deterministic geometric module for TPA measurement and saw blade size selection.

## 2. Materials and Methods

### 2.1. Image Acquisition and Preprocessing

A total of 200 lateral hindlimb radiographs from 130 dogs were retrospectively collected by five experienced surgeons at a single institution. All dogs had been diagnosed with cranial cruciate ligament rupture and were treated with various surgical procedures or rehabilitation orthoses. All images were obtained with the client’s informed consent and stored in the Digital Imaging and Communications in Medicine (DICOM) format. All patient-identifying information was fully anonymized prior to analysis in accordance with institutional data privacy protocols. The pixel spacing value documented in the DICOM header was used to convert pixel-level distances into millimeters (mm/px) for subsequent geometric calculations. Images with a Photometric-Interpretation attribute of MONOCHROME1 were intensity-inverted to standardize pixel polarity.

Each image was preprocessed into a three-channel input representation to provide the model with complementary radiographic information, rather than enforcing a single-contrast representation across all landmarks. Channel 1 consisted of the raw grayscale image, normalized to the range zero to one, preserving the native global signal pattern and cortical bone contrast. Channel 2 was generated using Contrast Limited Adaptive Histogram Equalization (CLAHE) with a clip limit of 3.0 and a tile grid size of 8 × 8, which locally enhances the visibility of cortical boundaries and plateau contours that may be obscured by uniform contrast adjustment [28,29]. Channel 3 applied power-law (gamma) correction with γ = 1.5 to increase overall image brightness, thereby emphasizing intermediate-intensity structures that may be difficult to distinguish in routine clinical radiographs [30].

A square isotropic region of interest (ROI) was cropped for each case, centered on the bounding box encompassing the five anatomical landmarks, with a uniform padding of 220 pixels applied to all sides. The longer axis of the padded bounding box was utilized to determine the side length of the square, which was subsequently resampled to 384 × 384 pixels using bicubic interpolation [31]. This approach standardizes the spatial context presented to the model, thereby ensuring a consistent input scale regardless of the original image dimensions or aspect ratio.

### 2.2. Landmark Definition and Annotation

**Figure 1:**
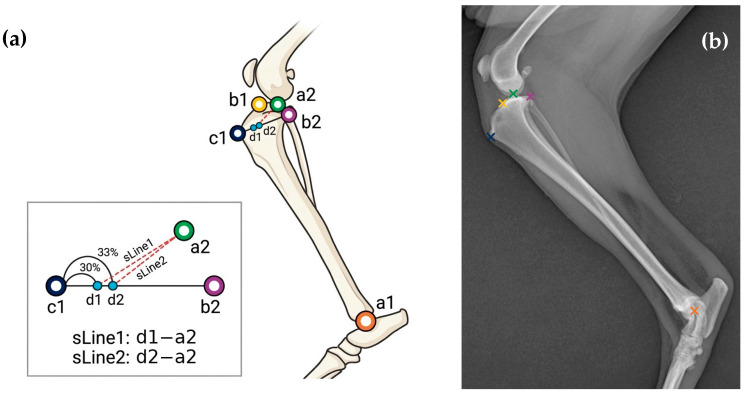
Anatomical landmark positions and geometric structure overlaid on a lateral hindlimb illustration (**a**) and radiograph (**b**). Each landmark is color-coded: tibial functional axis (a1–a2), tibial plateau line (b1–b2), and tibial tuberosity reference point (c1). The magnified inset illustrates the geometric construction used to estimate saw size, showing the distances from a2 to reference points d1 and d2 along the c1–b2 line segment (sLine1 and sLine2, respectively).

Five anatomical landmarks were identified on lateral hindlimb radiographs to enable the geometric calculations necessary for TPLO surgical planning. The anatomical locations and definition of each landmark are summarized in Table 1, and their geometric relationships are illustrated in Figure 1. All landmark coordinates were manually annotated by a single board-certified veterinary surgeon with expertise in orthopedic surgery (T.K.) and stored as two-dimensional pixel coordinates (x, y) in JSON format. The tibial functional axis was defined as the line segment connecting a1 (distal tibial joint center) and a2 (intercondylar eminence), and the tibial plateau axis was defined as the line segment connecting b1 (cranial point of the tibial plateau) and b2 (caudal point of the tibial plateau). The landmark c1 (tibial tuberosity reference point) served as a geometric anchor for saw size estimation. To ensure directional consistency across cases, automated coordinate normalization was applied before all calculations, ensuring that a2 is always proximal to a1 and that b2 is distal and caudal to b1, thereby preventing orientation-dependent errors arising from image-acquisition variability.

### 2.3. Model Architecture and Training

**Figure 2:**
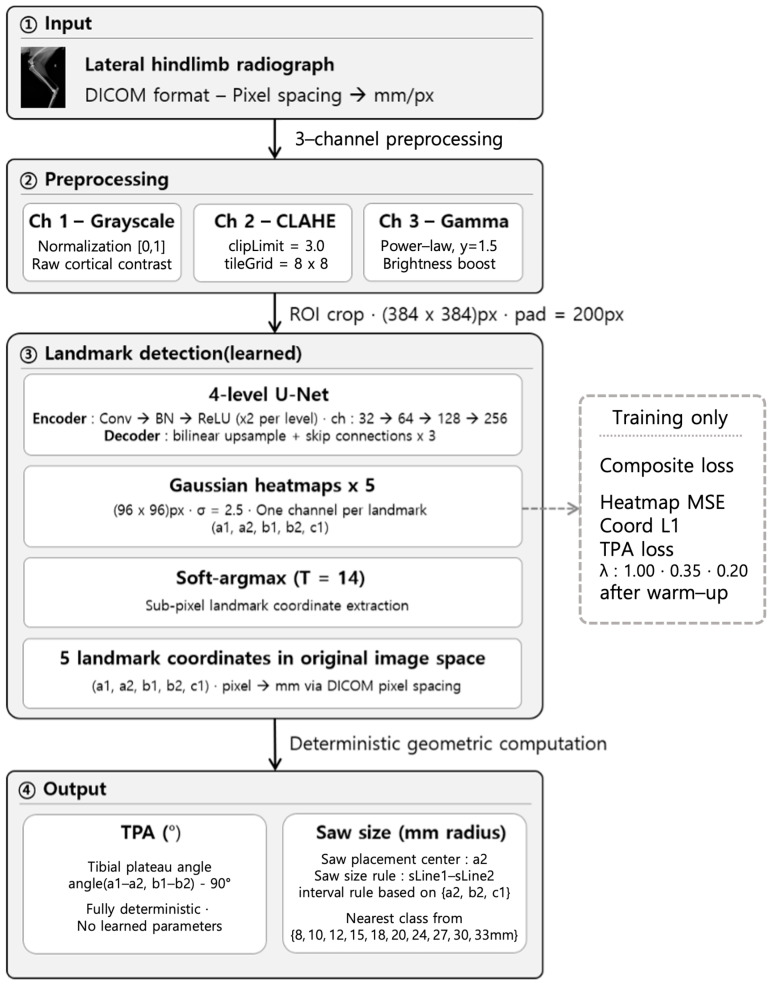
Overview of the fully automated pipeline. Lateral hindlimb radiographs in Digital Imaging and Communications in Medicine (DICOM) format are preprocessed into three complementary input channels (normalized grayscale, Contrast Limited Adaptive Histogram Equalization (CLAHE)-enhanced, and gamma-corrected). A square region of interest (ROI) centered on the tibial anatomy is extracted and resampled to 384 × 384 pixels. The four-level U-Net predicts Gaussian heatmaps for five landmarks, from which sub-pixel coordinates are extracted via soft-argmax. tibial plateau angle (TPA) and saw size are subsequently derived from the predicted landmark coordinates through deterministic geometric computation, without any additional learned parameters.

The fully automated pipeline described in this section is schematically illustrated in Figure 2. A customized four-level U-Net convolutional neural network (CNN) was developed for automated detection of anatomical landmarks. U-Net is an encoder–decoder architecture widely used in medical image segmentation and coordinate detection tasks, owing to its ability to combine high-level semantic features extracted by the encoder with fine-grained spatial information recovered through skip connections in the decoder [31]. The encoder consisted of four successive downsampling stages with channel dimensions of 32, 64, 128, and 256, respectively. Each stage consisted of two consecutive blocks of convolution → batch normalization → rectified linear unit (ReLU) activation [32], followed by max pooling. The decoder used bilinear upsampling with three skip connections to progressively restore spatial resolution.

The model’s output consisted of five Gaussian heatmaps of size 96 × 96 pixels (one per landmark, σ = 2.5). Each heatmap represents the probability distribution of a landmark’s location as a two-dimensional Gaussian centered at the annotated coordinate. The output resolution was intentionally set lower than the input resolution (384 × 384 pixels) to reduce computational cost while maintaining sufficient spatial precision for landmark localization. Sub-pixel landmark coordinates were subsequently extracted from the predicted heatmaps using differentiable soft-argmax, which computes a spatially weighted expectation over the probability distribution instead of selecting the discrete maximum-response location (argmax). This approach yields continuous-valued coordinate predictions and supports stable gradient-based optimization during training [33]. A temperature scaling parameter (T = 14) was applied to the heatmap logits prior to softmax normalization to control the sharpness of the probability distribution: higher values of T concentrate probability mass around the peak, thereby enhancing coordinate precision, whereas excessively large values may destabilize training. The value T = 14 was selected through preliminary experiments to balance localization sharpness and training stability. Extracted coordinates were subsequently back-transformed into the original image coordinate space via the inverse of the affine transformation applied during ROI cropping.

The model training utilized a composite loss function combining three terms. The initial term was a heatmap mean-squared error (MSE) loss (λ = 1.00), which penalizes the pixel-wise discrepancy between the predicted and ground-truth Gaussian heatmaps. The second term involved a weighted coordinate L1 loss (λ = 0.35), which directly minimizes the absolute positional error of each predicted landmark coordinate and compensates for cases where heatmap-level supervision alone is insufficient to resolve fine coordinate-level discrepancies. The third term involved a geometry-aware TPA loss (λ = 0.20), which penalizes the absolute angular difference between the TPA derived from the predicted landmarks and the reference TPA, thereby incorporating clinically significant angular accuracy directly into the training objective. To prevent training instability caused by unstable landmark predictions in early epochs, the TPA loss was implemented only after 10 warm-up epochs. During this initial phase, the model primarily focused on learning anatomically plausible landmark positions prior to further optimization for the derived clinical angle. This curriculum-like strategy is especially crucial for landmark detection tasks, where minor positional errors at multiple landmark sites can have asymmetric effects on downstream geometric measurements [34].

The full dataset of 200 cases was randomly split into training (80%; *n* = 160) and validation (20%; *n* = 40) sets using a fixed random seed (seed = 42) to ensure reproducibility. Because radiographs were collected from 130 individual dogs and some dogs contributed more than one radiograph, it is possible that images from the same dog were distributed across both the training and validation sets. Specifically, dogs with bilateral stifle involvement contributed one radiograph per limb (left and right acquired separately), whereas dogs with unilateral involvement contributed a single radiograph; no dog contributed repeated radiographs of the same limb. Although a strict dog-level split was not applied, each radiograph represents an independent acquisition event with its own landmark annotation and pixel-level variability; the split was performed randomly with a fixed seed (seed = 42). Future studies should implement dog-level data partitioning to eliminate this potential source of optimistic bias.

### 2.4. Geometric Computation of TPA and Saw Size

Both TPA and saw size were obtained from the predicted landmark coordinates via fully deterministic geometric computations, without utilizing any supplementary learned parameters. This design guarantees complete transparency and reproducibility in the measurement process: given identical landmark coordinates, the pipeline will invariably generate identical outputs, thereby offering a level of clinical interpretability that purely end-to-end learned regression approaches are unable to provide.

#### 2.4.1. TPA Computation

TPA is defined as the angle between the plateau axis and the line perpendicular to the tibial functional axis. Specifically, the angle between the tibial functional axis (a1–a2) and the tibial plateau line (b1–b2) was computed from the dot product of their unit direction vectors, and the TPA was obtained as the absolute difference between this angle and 90°. When the computed angle exceeded 90°, a supplementary correction (180° − angle) was applied to ensure that TPA values consistently ranged from 0° to 90°.

#### 2.4.2. Saw Size Computation

The estimation of the saw size was conducted in accordance with the geometric construction illustrated in Figure 1. Two reference points, d1 and d2, were established along the line segment connecting c1 and b2, located at distances representing 30% and 33%, respectively, of the total segment length from c1. The distances from a2 to d1 (sLine1) and from a2 to d2 (sLine2) were subsequently calculated and converted to millimeters utilizing the DICOM pixel spacing value. The predicted saw size was determined by finding the available or closest saw size class selected among a set of {8, 10, 12, 15, 18, 20, 24, 27, 30, 33} mm within the interval [sLine1, sLine2]. If there is no saw class in this interval, the class closest to the midpoint of interval [sLine1, sLine2] is selected. In cases of equal distance between two candidate classes, the option with the smaller value was selected. The saw placement center was fixed at landmark a2 (intercondylar eminence).

### 2.5. Evaluation Metrics

The performance of the model was evaluated across three domains: TPA prediction accuracy, saw size prediction accuracy, and landmark detection accuracy.

#### 2.5.1. TPA Evaluation

The primary metric for the accuracy of TPA prediction was the mean absolute error (MAE). Two accuracy thresholds were reported: 4.8°, the standard for variation between observers [2,3,4,5,6], and 2°, a stricter standard for variation between radiation imaging postures [2,6]. To quantify the linear agreement between predicted and reference TPA values, Pearson’s correlation coefficient (r) and the intraclass correlation coefficient (ICC(2,1)) were computed [35]. Systematic measurement bias and overall agreement were further characterized using Bland–Altman analysis, from which the mean bias and 95% limits of agreement (LoA) were derived [36]. Sensitivity and specificity for detecting clinically significant TPA measurement error (defined as absolute error > 4.8°) were additionally calculated.

#### 2.5.2. Saw Size Evaluation

The accuracy of saw size prediction was evaluated using three complementary metrics: exact match accuracy, within-one-class accuracy, and MAE. The within-one-class accuracy was included as a clinically relevant supplementary metric because using an adjacent saw size is generally considered an acceptable outcome in clinical practice.

#### 2.5.3. Landmark Detection Accuracy

Landmark detection accuracy was quantified for each landmark individually by computing the Euclidean distance between predicted and reference coordinates in millimeter units. The mean, median, standard deviation (SD), and maximum detection error were reported for each landmark. All statistical analyses were performed with Python (version 3.11.9; Python Software Foundation, Wilmington, DE, USA), and statistical significance was set at *p* < 0.05.

## 3. Results

### 3.1. Dataset Characteristics

The dataset comprised 200 radiographs from 130 dogs representing 14 breeds. The breed distribution was as follows: *Maltese* (*n* = 57, 28.5%), *Poodle* (*n* = 32, 16.0%), *mixed-breed* (*n* = 29, 14.5%), *Yorkshire Terrier* (*n* = 16, 8.0%), *Maltipoo* (n = 11, 5.5%), *Chihuahua* (*n* = 11, 5.5%), *Welsh Corgi* (*n* = 10, 5.0%), *Pomeranian* (*n* = 10, 5.0%), *Bichon Frisé* (*n* = 9, 4.5%), *Cocker Spaniel* (*n* = 4, 2.0%), *Miniature Pinscher* (*n* = 4, 2.0%), *Spitz* (*n* = 4, 2.0%), *Labrador Retriever* (*n* = 2, 1.0%), and *Shih Tzu* (*n* = 1, 0.5%). Body weight ranged from 2.4 to 38.0 kg (mean 6.4 ± 4.1 kg). Based on body weight, 99 cases (49.5%) involved small-breed dogs (≤5 kg; mean body weight 3.8 ± 0.6 kg), 99 cases (49.5%) involved medium-breed dogs (5–15 kg; mean body weight 8.4 ± 2.2 kg), and 2 cases (1.0%) involved large-breed dogs (>15 kg; mean body weight 36.5 ± 2.1 kg). No cases were excluded on the basis of outlier screening for the primary analysis. Mean reference TPA was 32.08 ± 4.32°, and mean predicted TPA was 31.69 ± 4.08°.

### 3.2. Landmark Detection Performance

As shown in Table 2, landmark localization was generally strong, and the median error was below 1 mm for all five landmarks. The most notable pattern was the divergence between mean and median error for a1 and a2, indicating that most cases were localized accurately but that a small number of large failures substantially inflated the mean error for the axis landmarks.

**Figure 3:**
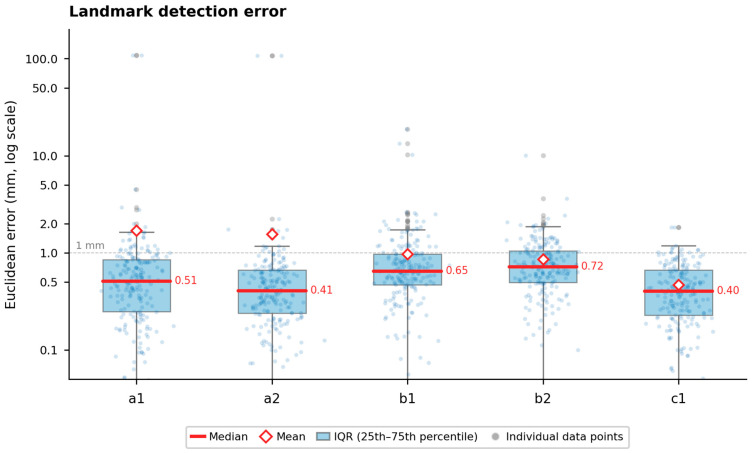
Distribution of per-landmark Euclidean detection error (mm, log scale). The solid red line indicates the median; the red diamond indicates the mean; the shaded blue box represents the interquartile range (25th–75th percentile); individual data points are overlaid as semi-transparent dots. The dashed horizontal line indicates the 1 mm reference threshold.

By contrast, c1 was consistently stable, and the plateau landmarks b1 and b2 showed comparatively small mean and median errors with shorter error tails than a1 and a2 (Figure 3). This distribution pattern suggests that extreme axis landmark failures can disproportionately distort the reconstructed tibial functional axis and therefore have a larger downstream effect on derived TPA.

### 3.3. TPA Prediction Performance

**Figure 4:**
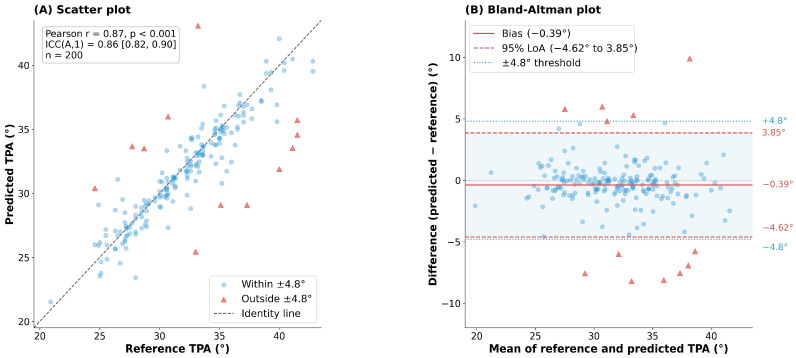
TPA prediction performance across all 200 cases. (**A**) Scatter plot of reference versus predicted TPA values. Cases within the ±4.8° threshold are shown as filled circles (●); cases exceeding this threshold are shown as filled triangles (▲) to aid identification in black-and-white print. The dashed line represents the line of perfect agreement (identity line). Pearson r, intraclass correlation coefficient (ICC(2,1)), and sample size are reported in the inset. (**B**) Bland–Altman plot of the difference between predicted and reference TPA values against their mean. The solid red line indicates the mean bias; the dashed red lines represent the 95% limits of agreement (LoA); the dashed blue lines indicate the ±4.8° clinically acceptable threshold.

**Figure 5:**
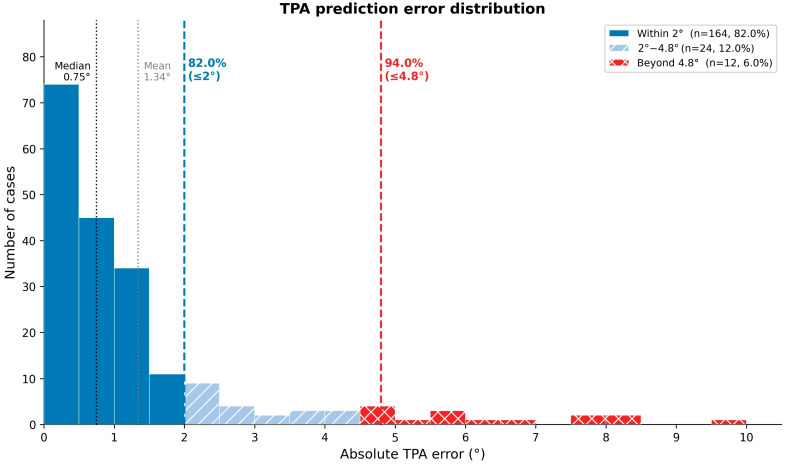
Distribution of absolute TPA prediction error across all 200 cases. Dark blue solid bars (■) indicate cases within ±2°; light blue hatched bars (▨) indicate cases in the 2–4.8° range; red cross-hatched bars (▩) indicate cases exceeding ±4.8°. Different fill patterns are provided to facilitate interpretation in black-and-white print. The dashed blue vertical line marks the ±2° threshold; the dashed red vertical line marks the ±4.8° clinically acceptable threshold. The dotted black and grey vertical lines indicate the median (0.75°) and mean (1.34°) absolute errors, respectively.

As summarized in Table 3 and illustrated in Figure 4 and Figure 5, the mean absolute error for TPA prediction was 1.34 ± 1.73°, and the median absolute error was 0.75°. Of 200 cases, 164 (82.0%) were within 2° of the surgeon reference and 188 (94.0%) were within 4.8°.

In the Bland–Altman analysis [36], the mean bias was −0.39°, and the 95% limits of agreement ranged from −4.62° to 3.85°. The Pearson correlation coefficient was 0.87. Across the entire cohort, the root mean square error was 2.19°, ICC(2,1) was 0.865 [35], and Lin’s concordance correlation coefficient was 0.864 [37]. For the detection of clinically significant TPA error (>4.8°), the sensitivity was 100% (12/12 threshold-exceeding cases correctly identified) and the specificity was 94.0% (188/200 within-threshold cases correctly classified as acceptable).

Overall, the distribution of errors was centered near zero, with most predictions clustered tightly around the surgeon reference. A minority of larger discrepancies persisted. The combination of a low median absolute error and broader agreement limits indicates that typical cases were accurately measured, while occasional significant deviations warrant the implementation of explicit review safeguards.

### 3.4. Saw Blade Size Prediction

As summarized in Table 4, saw blade size prediction was exactly correct in 175 of 200 cases (87.5%), and all predictions remained within one adjacent clinical class. Mean absolute error was 0.32 ± 0.85 mm, and the median absolute error was 0.00 mm.

Disagreement was bidirectional rather than unidirectional. The model predicted a larger saw size than the reference in 7 cases and a smaller size in 18 cases, suggesting that most disagreements occurred near category boundaries rather than reflecting a strong systematic bias.

## 4. Discussion

This study demonstrated that a hybrid TPLO planning pipeline, combining a learned landmark detector with deterministic geometric rules, can generate clinically meaningful outputs from routine lateral radiographs. The key message is not that every component performed uniformly well, but that strong cohort-level performance coexisted with a small number of clinically important landmark failures. This distinction is important because average accuracy alone can obscure the practical significance of rare but high-impact geometric errors.

These findings should be interpreted within the broader expansion of artificial intelligence in veterinary imaging. Recent studies have reported clinically relevant performance for thoracic radiograph classification [12], cardiomegaly detection [13,14], left atrial enlargement screening [15], pulmonary edema assessment [16], stifle disease diagnosis [17], tracheal collapse grading [18], elbow dysplasia screening [19], and automated hip-angle analysis [20,21,22]. The present work extends this trend to a more structured orthopedic measurement task by integrating landmark localization, derived geometry, and categorical surgical decision support into a single interpretable workflow.

The TPA prediction results were encouraging when viewed against the published literature on manual measurement variability [2,3,4,5,6]. In the present study, the mean absolute error was 1.34°, and 94.0% of predictions fell within 4.8° of the surgeon reference. Agreement within 2° was achieved in 82.0% of cases. Taken together, these findings suggest that the proposed model reached a clinically meaningful level of performance for most cases and may serve as a useful decision-support or cross-checking tool [23,25]. At the same time, the persistence of a minority of larger disagreements indicates that the system should not yet be regarded as an error-free autonomous measurement engine.

Agreement metrics supported the same interpretation. ICC(2,1) was 0.865, and Lin’s concordance correlation coefficient was 0.864, indicating that the pipeline preserved both case ordering and absolute TPA values to a clinically useful extent [35,37]. Likewise, Bland–Altman analysis [36] showed a small mean bias of −0.39°, suggesting that the model did not demonstrate a strong systematic tendency toward overestimation or underestimation at the cohort level.

The results at the landmark level significantly aid in elucidating why a favorable overall TPA performance may coexist with a limited number of considerable failures. The median localization error persisted at a low level across all five landmarks, signifying that the majority of radiographs were handled accurately [9,10]. Nonetheless, infrequent failures at the axis-related landmarks a1 and a2 engendered heavy-tailed error distributions, with maximum errors exceeding 100 mm in both cases. These catastrophic failures are consistent with a known failure mode in heatmap-based landmark detection, in which the network generates a secondary activation peak at an anatomically plausible but incorrect location. For a1 and a2, ambiguity arising from soft-tissue overlay or non-standard positioning can direct the predicted coordinate to a remote secondary peak, causing the reconstructed tibial functional axis to rotate by a large angle and disproportionately amplify the downstream TPA error even when plateau landmarks b1 and b2 are accurately localized. Future deployment frameworks should incorporate automatic plausibility checks on axis length and orientation to flag such cases for mandatory surgeon review before any clinical action is taken. This pattern highlights an important practical point: in geometry-dependent planning tasks, occasional landmark failures can exert a disproportionate effect on the final surgical measurement [1,8,27].

The points b1 and b2, which represent plateau points, are also worth considering. Their error distribution was more concentrated than that of a1 and a2, yet they directly influence the plateau line used in the TPA calculation [2,3,4,5]. Given the proximity of these two points, even a small coordinate disturbance can rotate the plateau and change the resulting angle. However, within this study group, such errors appeared to indicate limited but persistent ambiguity in the plateau contour rather than instances of rare, severe mislocalization.

The prediction of saw blade size demonstrated encouraging results. A complete agreement was observed in 87.5% of instances, with all predictions confined within one adjacent clinical class. In addition, disagreements occurred bidirectionally, indicating that the rule was not significantly biased towards overestimation or underestimation. This observation implies that most residual discrepancies likely occurred near boundary categories, where minor variations in landmark positioning could shift a case from one size class to another [7,8]. In this context, the algorithm should be considered as a consistent initial recommendation that diminishes the decision-making scope, rather than replacing the surgeon’s professional judgment.

The potential influence of breed conformation and body size on model performance warrants specific discussion. In the present dataset, 14 breeds were represented across a body weight range of 2.4 to 38.0 kg. Small-breed dogs (≤5 kg, *n* = 99) and medium-breed dogs (5–15 kg, *n* = 99) each accounted for 49.5% of cases, while only two large-breed cases were available (Labrador Retrievers, >15 kg). TPA mean absolute error was 1.28° for small breeds, 1.41° for medium breeds, and 1.05° for the two large-breed cases, with no meaningful difference across groups given the limited large-breed sample. Saw blade exact match accuracy was 89.9% for small breeds and 84.8% for medium breeds. Cases exceeding the 4.8° TPA threshold were equally distributed between small-breed (*n* = 6) and medium-breed (*n* = 6) dogs and did not cluster within specific breeds. Hypothetically, performance in breeds with markedly atypical tibial morphology—such as chondrodystrophic breeds (e.g., Bulldogs, Basset Hounds) or giant breeds—remains untested and could be adversely affected by distributional mismatch relative to the training data. External validation in more morphologically diverse populations will be essential.

A significant advantage of the current study lies in the hybrid architecture itself. Many end-to-end regression models generate output values without providing explicit explanations for their accuracy or inaccuracies. Conversely, the present methodology constrains the neural network to the task of anatomical landmark localization, while the final TPA and saw size outputs are retained as explicit geometric outcomes derived from the predicted coordinates. This separation improves interpretability, streamlines error analysis, and better aligns with clinical workflows, in which surgeons prefer to scrutinize the geometric basis of a recommendation prior to its acceptance [10,11,23,25]. Because the final outputs are derived from explicit coordinates, a future deployment framework could verify whether landmark positions are anatomically plausible, whether axis lengths or plateau widths conform to expected ranges, or whether minor perturbations of a specific point significantly influence the final recommendation. Such safeguards are a natural extension of a landmark-to-geometry workflow [11,38].

From a clinical implementation perspective, the most realistic near-term role of this system is supervised assistive use rather than fully autonomous planning [11,23,25]. A practical interface could automatically display the five landmarks, the reconstructed tibial axis and plateau line, the derived TPA, and the recommended saw size, while prompting focused surgeon review when confidence is low or geometric inconsistency is detected [11,38]. Such a workflow could reduce repetitive manual point placement while preserving human oversight and improving inter-user consistency [6,23].

Several limitations should be acknowledged. First, this was a retrospective single-center study conducted at a single institution, and the generalizability of the results to different practice settings, imaging protocols, and breed distributions remains uncertain [23,25]. A formal a priori sample size calculation was not performed prior to this retrospective study, as prospective power analysis for deep learning landmark detection tasks is methodologically challenging. The present dataset of 200 radiographs (160 training, 40 validation) should therefore be considered a proof-of-concept cohort, and future prospective studies should incorporate formal sample size planning to support definitive clinical validation. Second, and critically, the reference standard was based on annotations from a single expert rather than on a multi-reader consensus panel or repeated independent annotations [2,3,4,5,6]. Single-annotator reference standards introduce unknown observer bias and preclude direct estimation of the model’s performance relative to the true clinical gold standard; future studies should employ multi-reader annotation protocols to establish a more robust reference. Third, evaluation was limited to two-dimensional radiographs, whereas computed tomography may provide additional geometric information in more complex cases [7]. Fourth, the study focused on offline measurement accuracy rather than on prospective workflow integration, time savings, edit frequency, or postoperative clinical outcomes. Furthermore, the absence of external validation means that the generalizability of the reported performance metrics to different institutions, imaging protocols, and breed populations remains unconfirmed; multicenter external validation is an essential prerequisite before clinical deployment can be justified [11,23].

## 5. Conclusions

This study developed and validated a deep learning-based pipeline for automated TPA measurement and saw blade size prediction in canine TPLO planning. The proposed method showed clinically meaningful performance, with TPA errors remaining within the commonly reported range of interobserver variability in most cases and saw blade size prediction remaining within one adjacent clinical class across all 200 radiographs.

By combining a learned landmark detector with a deterministic geometric module, the proposed hybrid framework automated a key subjective step in TPLO planning while preserving interpretability and traceability of the final measurements. The study population encompassed 14 breeds spanning a body weight range of 2.4 to 38.0 kg, and performance metrics were broadly consistent between small- and medium-breed subgroups.

However, this study also has several limitations. It was a retrospective single-center study, the reference standard was based on annotations from a single expert, a formal a priori sample size estimation was not performed, and evaluation was limited to two-dimensional radiographs. In addition, the study did not assess prospective workflow integration, time savings, or postoperative clinical outcomes.

Therefore, future work should include multicenter external validation, prospective reader studies, and strategies for automatically flagging low-confidence cases for surgeon review. Such efforts will be essential to clarify the generalizability, safety, and real-world clinical utility of the proposed approach.

## Figures and Tables

**Table 1:** Definition and role of the five anatomical landmarks used in this study.

Landmark	Anatomical Location	Definition
a1	Distal tibial joint center	Defines the tibial functional axis (distal point)
a2	Intercondylar eminence	Defines the tibial functional axis (proximal point); saw placement center
b1	Cranial point of the tibial plateau	Defines the tibial plateau line
b2	Caudal point of the tibial plateau	Defines the tibial plateau line
c1	Tibial tuberosity reference point	Geometric reference point for saw size calculation

**Table 2:** Landmark localization error across the full dataset (*n* = 200).

Landmark	Description	Mean (mm)	Median (mm)	SD (Standard Deviation)/Max (mm)
a1	Distal tibial joint center	1.69	0.51	10.72/107.99
a2	Intercondylar eminence	1.56	0.41	10.70/107.72
b1	Cranial tibial plateau point	0.97	0.65	1.76/18.86
b2	Caudal tibial plateau point	0.86	0.72	0.82/10.05
c1	Tibial tuberosity reference point	0.47	0.40	0.31/1.83

**Table 3:** Summary of TPA prediction performance.

Metric	Value
Valid cases	200
Reference TPA, mean ± SD (degrees)	32.08 ± 4.32
Predicted TPA, mean ± SD (degrees)	31.69 ± 4.08
Mean absolute error (degrees)	1.34 ± 1.73
Median absolute error (degrees)	0.75
Within 2 degrees	164 (82.0%)
Within 4.8 degrees	188 (94.0%)
Mean bias, predicted − reference (degrees)	−0.39
95% limits of agreement (degrees)	−4.62 to 3.85
Pearson correlation coefficient	0.87
Root mean square error (degrees)	2.19
ICC(2,1)	0.865
Lin’s concordance correlation coefficient	0.864
Sensitivity for detecting TPA error > 4.8°	100% (12/12)
Specificity for cases within 4.8°	94.0% (188/200)

**Table 4:** Saw blade size prediction according to reference size.

Reference Size (mm)	Total Cases	Exact Match	AI Larger	AI Smaller
10	16	14 (87.5%)	2 (12.5%)	0 (0.0%)
12	88	79 (89.8%)	2 (2.3%)	7 (8.0%)
15	60	54 (90.0%)	1 (1.7%)	5 (8.3%)
18	26	19 (73.1%)	2 (7.7%)	5 (19.2%)
20	8	7 (87.5%)	0 (0.0%)	1 (12.5%)
27	2	2 (100.0%)	0 (0.0%)	0 (0.0%)

## Data Availability

The data and code supporting the findings of this study may be made available by the corresponding author upon reasonable request, subject to institutional and privacy restrictions.
